# Signatures of malaria-associated pathology revealed by high-resolution whole-blood transcriptomics in a rodent model of malaria

**DOI:** 10.1038/srep41722

**Published:** 2017-02-03

**Authors:** Jing-wen Lin, Jan Sodenkamp, Deirdre Cunningham, Katrien Deroost, Tshibuayi Christine Tshitenge, Sarah McLaughlin, Tracey J. Lamb, Bradley Spencer-Dene, Caroline Hosking, Jai Ramesar, Chris J. Janse, Christine Graham, Anne O’Garra, Jean Langhorne

**Affiliations:** 1 Malaria Immunology laboratory, Francis Crick Institute, 1 Midland Road, NW1 1AT London, United Kingdom; 2 Experimental Histopathology, Francis Crick Institute, 1 Midland Road, NW1 1AT London, United Kingdom; 3 Leiden Malaria Research Group, Leiden University Medical Center, Department of Parasitology, 2333 ZA Leiden, The Netherlands; 4 Immunoregulation and Infection laboratory, The Francis Crick Institute, NW1 1AT, London, United Kingdom

## Abstract

The influence of parasite genetic factors on immune responses and development of severe pathology of malaria is largely unknown. In this study, we performed genome-wide transcriptomic profiling of mouse whole blood during blood-stage infections of two strains of the rodent malaria parasite *Plasmodium chabaudi* that differ in virulence. We identified several transcriptomic signatures associated with the virulent infection, including signatures for platelet aggregation, stronger and prolonged anemia and lung inflammation. The first two signatures were detected prior to pathology. The anemia signature indicated deregulation of host erythropoiesis, and the lung inflammation signature was linked to increased neutrophil infiltration, more cell death and greater parasite sequestration in the lungs. This comparative whole-blood transcriptomics profiling of virulent and avirulent malaria shows the validity of this approach to inform severity of the infection and provide insight into pathogenic mechanisms.

According to the latest estimation by WHO, there were 214 million new cases of malaria worldwide in 2015 and 438,000 deaths (World Malaria Report 2015, www.who.int). Most of these deaths were due to complicated symptoms, such as cerebral malaria, severe malarial anemia, acute respiratory distress syndrome and abnormalities in blood coagulation (http://www.cdc.gov/malaria/about/disease. html). Both host and parasite factors influence clinical outcomes of malaria. In a study conducted in a *Plasmodium falciparum* and *P. vivax* infected population in Sri Lanka, only 30% of variations in disease severity could be explained by known factors, among which were prior exposure, host genetics and other host non-genetic factors^1^. The fact that 70% remained unexplained suggests a strong contribution of parasite virulence and its interaction with the host.

Despite decades of research, we do not know how the host responds to *Plasmodium* strains of different virulence, or how this leads to different levels of morbidity and mortality. In-depth analyses of immune responses to differently virulent parasites will likely reveal host factors that contribute to disease severity, and provide insight into mechanisms leading to immunoprotection or immunopathogenesis.

When investigating immune responses in infections or diseases in humans, the most relevant or important tissues are not usually readily accessible, which often limits these types of studies. Sampling of peripheral blood offers a feasible alternative as it is one of the “highways” of the immune system via which naïve, and activated or primed immune cells travel between lymphoid organs and the tissues affected by the infection. By profiling global transcriptomes of whole blood, insights can be obtained into the complex changes in systemic or even local host responses brought about by an infection, and thus inform more targeted mechanistic studies.

To investigate the use of genome-wide transcriptomic profiling of the whole blood in identifying pathology signatures in malarial infection, and to gain insights into the mechanisms underlying pathology, we used the well-establised *P. chabaudi chabaudi* rodent malaria model to study malarial immunology and pathology^2^,^3^. Using two strains of *P. c. chabaudi*, AS and CB, that differ in virulence in C57BL/6 mice, we performed high-resolution comparative whole-blood transcriptomic analysis throughout the acute phase of the blood-stage infection, and identified several transcriptomic signatures associated with severe malarial pathology before the onset of pathology or disease.

## Results

### The virulent CB strain of *P. c. chabaudi* induces more severe pathology in the acute phase of a blood-stage infection compared with the avirulent AS strain

**Figure 1:**
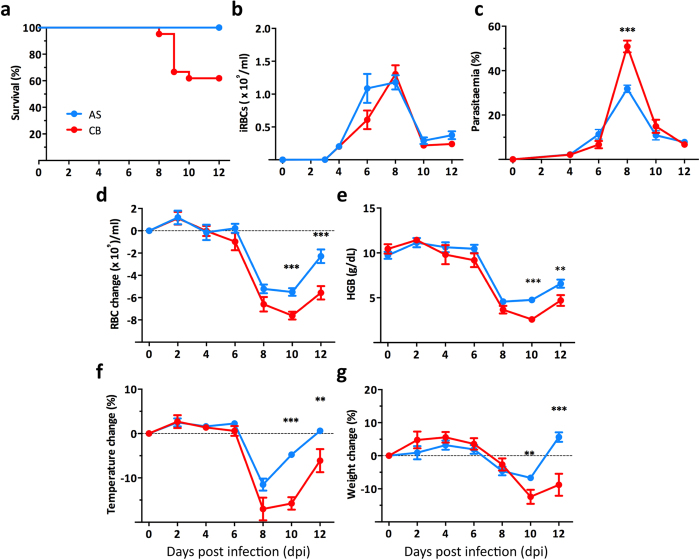
An infection with *P. c. chabaudi* CB parasites causes more severe pathology in the acute phase than infection with AS parasites in female C57BL/6 mice. **(a)** The survival rate of AS and CB infected mice during the acute phase of infection. **(b,c)** The total numbers of infected red blood cells (iRBC) per ml of blood (**b**) and the parasitemia (percentage of infected iRBC) (**c**) in the mice infected with AS or CB parasites. (**d,e)** The change in RBC numbers during the infection in AS or CB infected mice **(d)** and the hemoglobin (HGB) concentration in the blood of the infected mice (**e**). (**f,g)** The percentage change in temperature **(f)** and weight (**g**) during the infection in AS or CB infected mice. Data were pooled from 21 mice in 3 independent experiments. Graphs show mean with SEM, Mann-Whitney U test was performed (* p < 0.05, **p < 0.005, ***p < 0.0005).

Infection of C57BL/6 mice was initiated by intraperitoneal inoculation of 10^5^ infected red blood cells (iRBC) of *P. c. chabaudi* AS or CB. Infection with the CB strain gave rise to a more severe infection, resulting in 40% (range 20–60%) of mice reaching the humane end points (more than 25% weight loss, persistent laboured breathing and severe hypothermia), while all AS infected mice survived the infection without showing severe pathologies (Fig. 1a). AS and CB infected mice showed comparable iRBC loads (parasitemia multiplied by total RBC numbers), despite the fact that higher peak parasitemias were observed in the acute CB infection (Fig. 1b,c). A more severe RBC loss with a significantly lower hemoglobin concentration was observed in CB infection at 10 days post infection (dpi) compared to that in AS infected mice, agreeing with previous observations, which showed more severe anemia in CB infected BALB/c mice^4^. Moreover, the RBC loss in CB infected mice is longer lasting, even after the peak of infection at 12 dpi (Fig. 1d,e). In addition, at 10 dpi, CB infected mice showed greater temperature and weight loss (Fig. 1f,g).

### Virulent CB and avirulent AS strains of *P. c. chabaudi* induce distinct responses in host whole blood transcriptome

To investigate whether AS and CB parasites induce different host responses that might contribute to the differences in severity of the blood-stage infection we carried out genome-wide transcriptomic analyses of whole blood during the acute phase of infection. Peripheral blood was collected into Tempus tubes via cardiac puncture from C57BL/6 mice infected with AS or CB at 2, 4, 6, 8, 10, and 12 dpi. Blood samples collected from age-matched uninfected animals at day 0 and day 12 were used as naïve controls to exclude transcriptional changes due to time. The total RNA was extracted, depleted of globin mRNA and analysed using Illumina Mouse WG-6 v2.0 Beadarrays.

**Figure 2:**
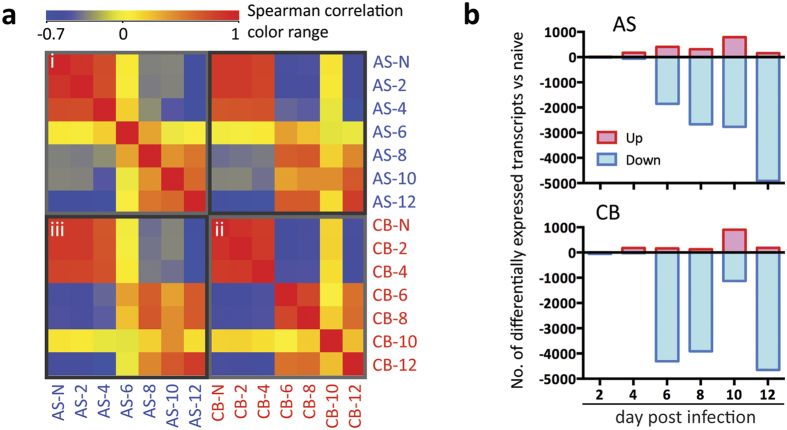
Whole blood transcriptomic analyses reveal distinct host responses in *P. c. chabaudi* avirulent AS and virulent CB infections. (**a**) Heatmap of the Spearman rank correlation matrix. The transcripts were normalised across the median of all samples. Colours represent Spearman rank correlation coefficient between each condition with red representing positive rank correlation level above zero (yellow) for a given condition pair, and blue representing negative rank correlation level. (i) correlation amongst AS infected samples; (ii) correlation amongst CB infection samples; (iii) correlation between samples from AS and CB infections. **(b)** Bar charts showing the number of transcripts that were differentially expressed compared to naïve controls in AS and CB infections at different time points. Differential expression analysis (ANOVA unequal variance with post-hoc HSD test, FDR < 0.01) was performed on transcripts normalised to the median of their respective naïve controls.

Spearman’s rank correlation coefficient (r_s_) analysis of unfiltered transcripts normalised across the median of all samples, revealed high levels of similarity amongst naïve and 2, 4 dpi samples in both AS and CB infections (Fig. 2ai,ii, r_s_ ranging from 0.73 to 0.88), while from 6 dpi onwards the whole blood transcriptomes diverge significantly from the earlier time points (r_s_ ranging from 0.08 to −0.59 compared to naïve controls). When comparing AS and CB infections at each of the time points, lower correlation values were observed between 6–10 dpi (Fig. 2aiii, r_s_ = 0.37, 0.67, 0.44, respectively). This indicates that AS and CB infections induce different host responses at these times of infection. AS infected mice and those CB infected mice that survived this phase of infection showed similar profiles at 12 dpi (Fig. 2aiii, r_s_ = 0.82).

Differential expression analysis (ANOVA unequal variance with post-hoc HSD test, FDR < 0.01) was performed on transcripts normalised to the median of their respective naïve controls (Supplementary Table 1). At any of the post-infection time points examined, there were 6226 differentially expressed transcripts with a greater than 2-fold change in the infected mice compared to the naïve mice (Fig. 2b, Supplementary Fig. 1a). Consistent with correlation analyses of unfiltered transcripts (Fig. 2a), only a few transcripts were differentially expressed at 2 and 4 dpi (Fig. 2b). Strikingly, at 6 dpi, a large majority of transcripts were down-regulated compared to naïve controls in both AS and CB infections. However, the number was greater in CB infected mice compared to AS (AS 1856 out of 2265 transcripts, CB 4306 out of 4471 transcripts) (Fig. 2b). The number of down-regulated transcripts increased steadily from 6 to 12 dpi in AS infection (Fig. 2b). While down-regulation of transcripts was also observed at 6–8 dpi in the CB infection, the level of down-regulation was significantly lower at 10 dpi and there was a sudden up-regulation of transcripts occurred only in CB infected mice at this time point (Fig. 2b, Supplementary Fig. 1a), a critical time point when infected animals were either recovering from acute infection or suffering from severe pathologies leading to death (Fig. 1), indicating that these up-regulated transcripts may relate to the manifestation of severe pathology in the CB infection. Indeed this higher level of transcriptional up-regulation was further confirmed in 4 CB infected mice that had reached humane end points at 9 dpi (Supplementary Fig. 1b). This up- or down-regulation of gene expression in whole blood are due to both the leukocyte population changes and transcript regulation during the infection (Supplementary Fig. 1c).

### Hierarchical clustering and modular transcriptome analyses identified biological processes in the blood associated with severe pathology

**Figure 3:**
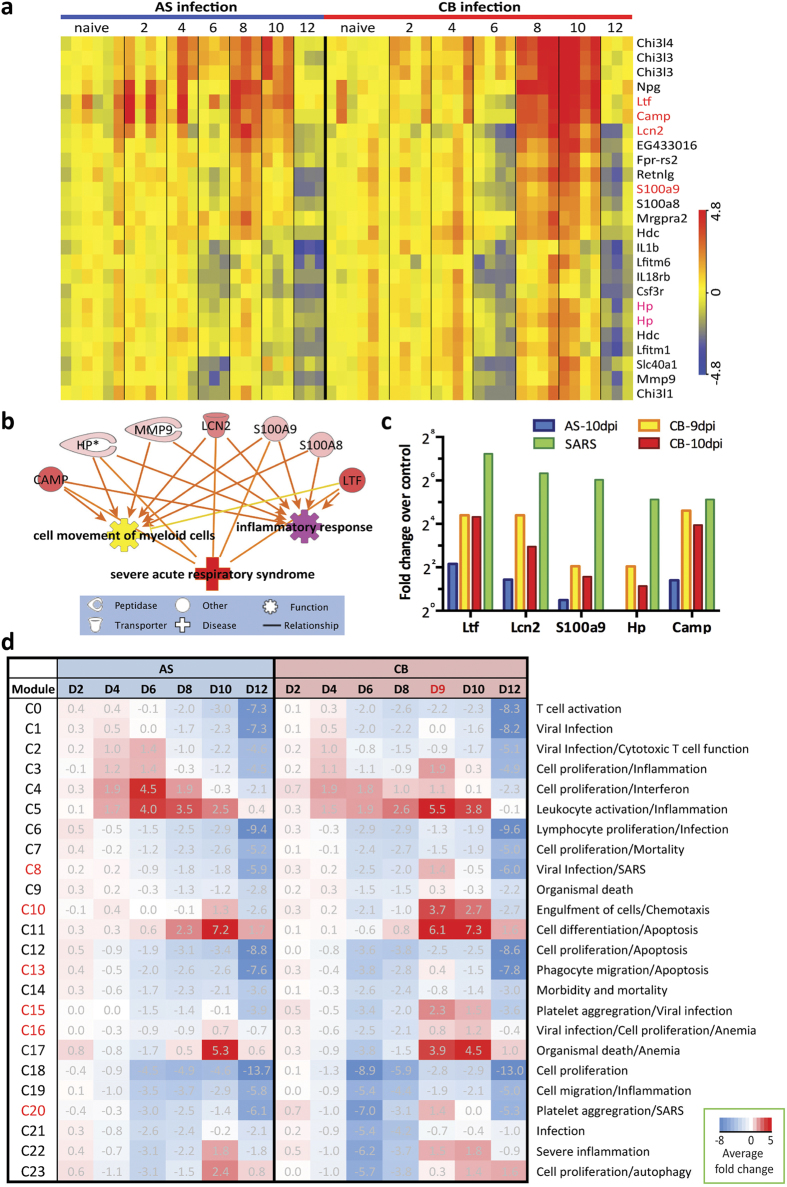
Hierarchical clustering and modular analyses reveal biological processes associated with severe malarial pathology. (**a**) A set of probes representing 22 genes that were more highly up-regulated in CB than AS infection between 8–10 day post infection (dpi). They were identified from hierarchical clustering of all differentially expressed transcripts from any infected samples compared to naïve controls (related to Supplementary Fig. 1). Colour indicates the log 2 transformed normalized expression intensity. (**b**) Disease and Function analysis in IPA (Ingenuity Pathway Analysis) identified some of the 22 genes shown in (**a**) were significantly enriched in functions of ‘cell movement of myeloid cells’ and ‘inflammatory response’, and disease of ‘severe acute respiratory syndrome (SARS)’. (**c**) Five genes enriched in the SARS gene signature (red text in **a**) were even more highly up-regulated compared to naïve controls in the blood extracted from 4 CB infected mice that had reached humane end points at 9 dpi compared to randomly selected mice infected with AS or CB parasites at 10 dpi. Fold changes in SARS patients were from ref. 13. (**d**) The twenty-four modules identified by using self-organising map (SOM) method were presented with the average fold change of all differentially expressed transcripts within each module compared to naïve controls. Red represents positive mean fold change above zero (white) and blue indicates negative mean fold change. The clusters were annotated with IPA Diseases and Function analysis with manual curation. Clusters indicated in red showed greater up-regulation in CB infection during 9–10 dpi.

A hierarchical clustering of the 6226 differentially expressed transcripts of whole blood revealed 2 clusters of transcripts comprised of 22 genes that were significantly more up-regulated in CB infected mice between 8 and 10 dpi than in the blood of AS infected mice (Supplementary Fig. 2, 22 genes in Fig. 3a), during which severe clinical signs were manifested (severe hypothermia, weight loss and host death) (Fig. 1). Ingenuity Pathway Analysis (IPA) Diseases and Function analysis showed that some of these genes were significantly enriched in ‘functions’ of inflammation and myeloid cell movement (Fig. 3b). Interestingly, 5 genes (Fig. 3a, indicated in red) were enriched in ‘disease’ of severe acute respiratory syndrome (SARS) (Fig. 3b). These 5 genes have been shown to be amongst the top 10 up-regulated genes in transcriptomic analysis of PBMCs (peripheral blood mononuclear cells) isolated from SARS patients suffering from severe lung inflammation^5^ (Fig. 3c). Importantly, in 4 of the CB infected mice that had reached humane end points at 9 dpi, these 5 genes showed an even higher level of up-regulation compared to naïve controls (Fig. 3c), indicating possible lung pathology in these 4 CB infected mice.

To identify further pathology-related blood transcriptomic signatures, we included the microarray data of blood isolated from the 4 CB-infected mice that had reached the humane end points, and followed the same differential expression analysis as described above, yielding a total of 6321 differentially expressed transcripts (Supplementary Table 2). A self-organising map (SOM) clustering method was used to generate clusters of co-expressing transcripts (Supplementary Fig. 3, Supplementary Table 3)^6^. The cluster expression level was defined as the average fold-change of all transcripts within each module compared to that of naïve controls (Fig. 3d). The clusters were annotated with IPA Diseases and Function Analysis and manually curated. Several clusters, C8, C10, C13, C15, C16 and C20 showed greater up-regulation in CB infection during 9–10 dpi, indicating their association with lethality/severe pathology in CB infection (Fig. 3d, indicated in red).

### Modular analysis identified an early platelet aggregation signature only associated with the virulent *P. c. chabaudi* CB infection

**Figure 4:**
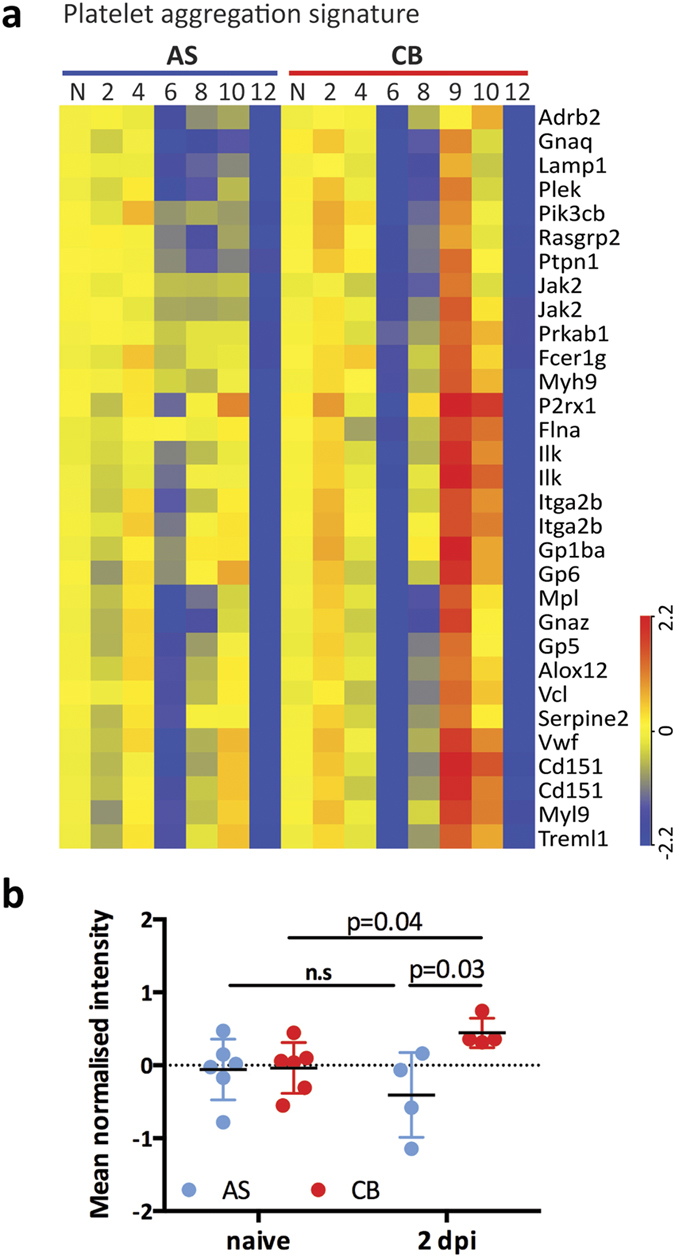
An early platelet aggregation signature in CB infection. (**a**) Expression heatmap of 27 genes associated with the platelet aggregation transcriptomic signature identified from modules C15 and C20. Colour indicates log 2 transformed normalized expression intensity. (**b**) The mean normalized intensity of these 27 genes was quantified in naïve and infected mice at 2 days post infection (dpi). Each dot represents an individual mouse. Mean and standard deviation are shown. Multiple T test was performed and the p values are provided, n.s indicates no significance.

Clusters C15 and C20, were exclusively up-regulated in the CB infection, especially in the mice that had reached humane end points at 9 dpi (Fig. 3d). C15 showed a maximum average of 2.3-fold up-regulation and C20 showed a maximum average of 1.4-fold up-regulation in CB infected mice that were dying from the infection. Twenty-seven genes within these 2 clusters are associated with platelet aggregation/pro-coagulation, a common feature of severe malaria infection^7^ (Fig. 4a). They are significantly enriched in canonical pathways Integrin Signalling (−log(P value) 4.3, Z score 2) and Actin Cytoskeleton Signalling (−log(P value) 4.2, Z score 2). Importantly, this set of genes was significantly up-regulated in all CB infected mice as early as 2 dpi (Fig. 4b), at the time of which very few genes were differentially expressed compared to naïve controls (Fig. 2b).

### Modular analysis identified a more pronounced and longer-lasting anemia signature in the virulent *P. c. chabaudi* CB infection

**Figure 5:**
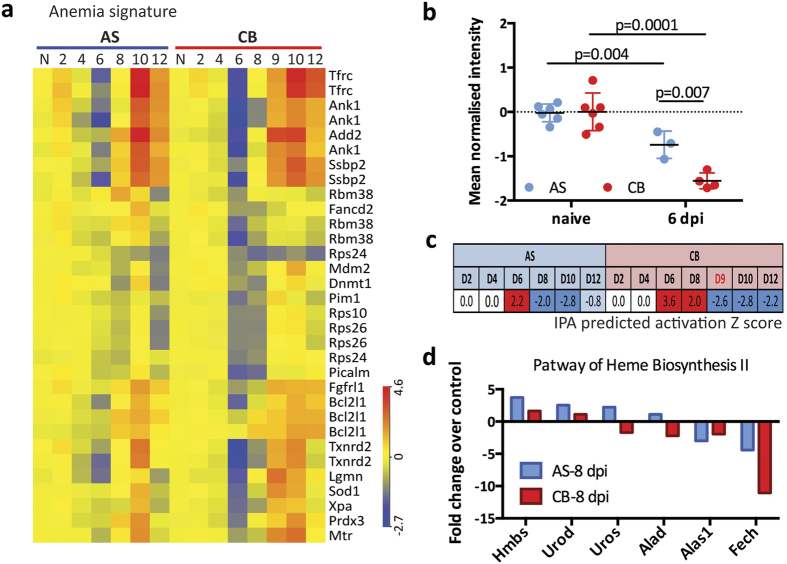
A more pronounced and longer-lasting anemia signature in CB infection. (**a**) Expression heatmap of 21 genes associated with the anemia transcriptomic signature identified from modules C16 and C17. Colour indicates log 2 transformed normalized expression intensity. **(b)** The mean normalized intensity of these 21 genes was quantified in naïve and infected mice at 6 days post infection (dpi). Each dot represents an individual mouse. Mean and standard deviation are shown. Multiple T test was performed and the p values are provided. **(c)** The predicated activation Z scores of anemia (by IPA Disease and Function analysis) showed significantly higher activation in CB infection at 6 dpi compared to AS infection and remained activated until 8 dpi. **(d)** Bar chart shows fold change (compared to naïve controls) of genes that are in the pathway of heme biosynthesis II and were differentially expressed in the blood of AS or CB infected mice.

Two clusters, C16 and C17 contained 21 genes related to anemia (Fig. 5a). For most of these genes, their down-regulation is associated with anemia, and up-regulation is associated with alleviation of anemia (Supplementary Fig. 4). The anemia signature was already present at 6 dpi in both infections (Fig. 5a), prior to clinical observation of RBC and hemoglobin loss at 8 dpi (Fig. 1). The mean normalised intensity of these 21 genes was significantly down-regulated compared to naïve controls, and CB infected mice had even significantly lower values (Fig. 5b). The peak activation Z scores calculated by IPA for this anemia signature of CB infection was 1.6-fold higher compared with that of AS (Fig. 5c, 3.6 vs 2.2, 6 dpi), agreeing with the more severe RBC loss (area under the curve analysis) in CB than AS infection (1.5-fold, 36.0 vs 23.5) (Fig. 1d). Interestingly, at 8 dpi, 13 out of the 21 genes within this signature were already up-regulated in AS infections compared to naïve controls; by contrast, the majority (15) of these genes were still down-regulated in the blood of CB infected mice (Fig. 5a, Supplementary Fig. 4). The activation Z score was −2.0 in AS infected mice indicating anemia alleviation at this time point, compared to that of 2.0 in CB infected mice (Fig. 5c), which indicates a longer-lasting anemia. This is consistent with the more severe RBC and hemoglobin loss in CB infection at 10 dpi compared to AS (Fig. 1d,e). Moreover, in support of this data, IPA Canonical Pathway Analysis of heme biosynthesis II pathway also showed that genes in this pathway were more down-regulated or less up-regulated in CB than in AS infected mice at 8 dpi compared to naïve controls (Fig. 5d). Together, these data suggest a deregulated or stressed erythropoietic response in CB infected mice, leading to a stronger and prolonged severe anemia (Fig. 1d,e).

### Lung inflammation signature in whole blood is confirmed with infiltration of neutrophils into the lungs of *P. c. chabaudi* CB infected mice

**Figure 6:**
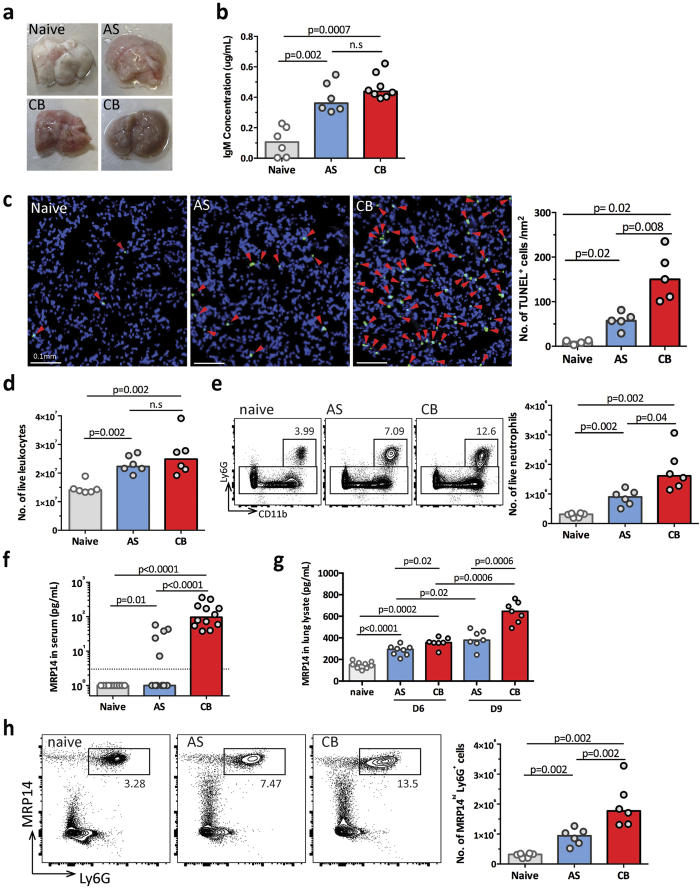
Higher level of neutrophil infiltration in the lungs of CB infected mice. **(a)** Representative images of *ex vivo* perfused lungs from naïve, AS and CB infected mice at 9 days post infection (dpi). **(b)** IgM concentration in the bronchoalveolar lavage fluid in the mice infected with AS or CB at 9 dpi compared to naïve mice. **(c)** Representative images of TUNEL-stained sections of perfused lungs from naïve, AS and CB infected mice at 9 dpi (left) and the bar chart showing the numbers of TUNEL^+^ cells (right, n = 4–5). Red arrowheads indicate TUNEL-positive cells. **(d)** Live leukocyte numbers in the lungs of AS or CB infected mice at 9 dpi compared to naïve controls. Results in (**b**,**d**) are representative of two independent experiments (n = 5–6 per experiment). **(e)** Representative FACS plots showing the increase of percentages of Ly6G^+^ CD11b^+^ neutrophils in the lungs of AS and CB infected mice compared to uninfected naïve controls (left), and the bar chart showing quantified neutrophil numbers (right). **(f)** The MRP14 protein concentration was quantified by ELISA in serum of naïve, AS and CB infected mice at 9 dpi. Dashed line indicates ELISA detection limit. Data were pooled from 2 independent experiments (n = 6 per experiment). **(g)** The MRP14 protein concentration was quantified by ELISA in lung lysate of naïve, AS and CB infected mice at 6 and 9 dpi. Data were pooled from 2 independent experiments (n > 6 per experiment). **(h)** Representative FACS plots showing the increase of percentages of MRP14^+^ Ly6G^+^ neutrophils in the lungs of AS and CB infected mice at 9 dpi compared to naïve controls (left), and the bar chart showing quantified MRP14^+^ Ly6G^+^ neutrophil numbers (right). In all bar charts, median values are shown and each dot represents an individual mouse. Mann-Whitney U test was performed, p values are provided and n.s indicates no significance.

In addition to the 5 genes we identified from hierarchical clustering, the SOM analysis revealed a further 18 genes in clusters C8 and C20 related to SARS, and the majority of these 18 genes were up-regulated only in CB infected mice that reached human end point at 9 dpi (Supplementary Fig. 5a). We therefore investigated whether this lung inflammation signature was reflected by more severe lung pathology in CB infected animals. Lungs isolated from systemically perfused CB infected animals were of a darker coloration than lungs of uninfected or AS infected mice (Fig. 6a). Examination of bronchoalveolar lavage fluid showed significantly higher levels of IgM in the lungs of mice infected with AS or CB parasites compared to naïve mice (Fig. 6b). The hematoxylin and eosin (H&E) stained sections of perfused lungs from both AS and CB infected mice showed signs of leukocyte infiltration. In the lungs of some CB infected mice, patches of dense leukocyte accumulation between epithelial walls were observed in close proximity to hemozoin (Hz) crystals (Supplementary Fig. 5b). We also observed more cell death in the lung tissues of CB infected mice (greater than two fold) compared to AS infected mice by TUNEL staining (Fig. 6c).

Flow cytometry analysis confirmed leukocyte infiltration in the lungs of both AS and CB infected mice compared to that in naïve mice (Fig. 6d, gating strategy in Supplementary Fig. 6). We further characterised the cell populations within the infiltrating leukocytes in the lungs of infected animals. Although CD3^+^ T cells, CD19^+^ B cells and CD3^−^CD19^−^ innate cell numbers all increased in both infections, they did not significantly differ between AS and CB infections; however, there was a trend towards higher T cell numbers in the AS infection, and more innate cells in the CB infection (Supplementary Fig. 6b,c). Within the innate cell populations, both the percentage and the cell numbers of Ly6G^+^ CD11b^+^ neutrophils were significantly increased in lungs of CB infected mice compared to AS infected mice (Fig. 6e), whereas other myeloid cell populations did not significantly differ between the two infections (Supplementary Fig. 6d).

This observation of neutrophil infiltration is consistent with the IPA ‘Disease and function’ analysis on the SARS signature, which was up-regulated more in CB infection compared to AS infection, indicating myeloid cell movement (Fig. 3b), especially neutrophils (Z score 2.182). One of the top up-regulated genes, S100A9 (MRP14, myeloid-related protein 14), indicates that neutrophils may be involved in the lung pathology of the CB infection. We therefore investigated whether the up-regulation of MRP14 transcript in the blood is associated with higher protein level and neutrophil infiltration in the lungs. We analysed the concentration of MRP14 protein in the serum at 9 dpi. While high level of MRP14 protein was also detected in sera of all (12) CB infected mice, it was below detection limit by ELISA in the sera from 5 out of 12 AS infected mice and all (12) from naïve mice (Fig. 6f). We next measured the amount of MRP14 in the whole lung lysates, and found that CB infected mice contained more than twice the amount of MRP14 protein compared to that of lungs of AS infected mice at 9 dpi; moreover, this upregulation was observed as early as 6 dpi (Fig. 6g). IFNg, IL6, KC (CXCL1) and LIX (CXCL5) were higher in the lungs of CB infected mice, indicating a heightened proinflammatory response in the CB infection (Supplementary Fig. 7a). Immunohistochemical staining of MRP14 on lung sections showed more MRP14^+^ cells present in the lungs of CB infected mice compared to naïve and AS infected mice (Supplementary Fig. 7b), and flow cytometry analysis confirmed a greater than two fold increase of MRP14^hi^Ly6G^+^ neutrophils in CB compared to AS infected mice (Fig. 6h).

Together, these data show that the blood transcriptomic signature of lung inflammation is linked to an MRP14-associated neutrophil response in the lungs of mice infected with the more virulent CB strain of *P. c. chabaudi* compared with the avirulent AS strain.

### Greater neutrophil infiltration in virulent *P. c. chabaudi* CB infection is associated with greater sequestration in the lungs

**Figure 7:**
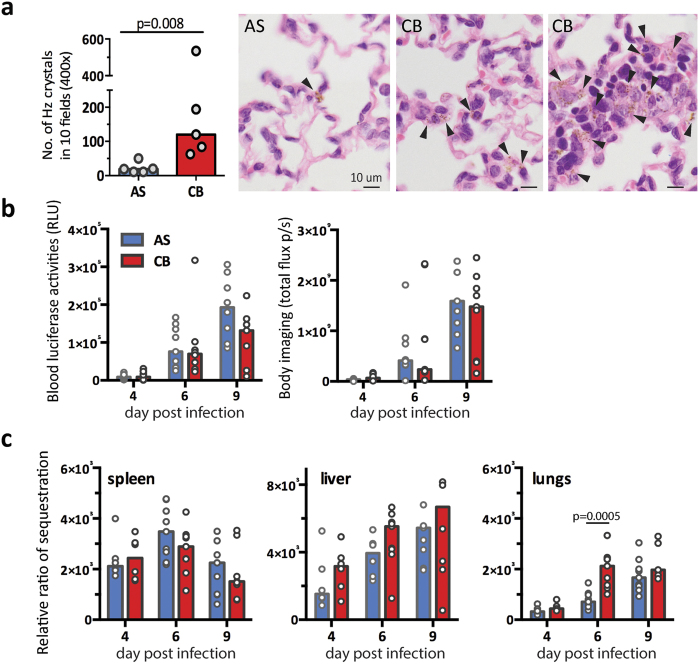
Greater sequestration and hemozoin deposition in the lungs of CB infected mice. **(a)** Higher number of hemozoin (Hz) crystals was observed in the perfused lungs of CB infected mice compared to AS at 9 dpi (left). The number of hemozoin crystals were quantified from 10 randomly selected fields on hematoxylin and eosin stained slides (400×). Representative images of Hz-containing lung sections (right). Black arrowheads indicate Hz crystals. **(b)** Total parasite load was determined by measuring luciferase activities in tail blood at late trophozoite stage (left, relative light units) or by whole body imaging (right, total flux per second). **(c)** Bar charts showing the relative ratio of sequestration in different organs, which was quantified as the level of luciferase activities in the perfused *ex vivo* organs relative to the total parasite load measured in peripheral blood at late trophozoite stage (**b**, left). All data in (**b**,**c**) were pooled from 2 independent experiments (n = 9–12 in total). In all bar charts, median values are shown and each dot represents an individual mouse. Mann-Whitney U test was performed, p values are provided when significant difference was observed.

Examination of H & E stained sections of perfused lung revealed a greater amount of Hz accumulated in the lungs of CB infected mice compared to AS infected mice (Fig. 7a). This observation suggested a higher level of sequestration/accumulation of iRBC in the lungs of CB infected mice. To investigate the level of sequestration of iRBCs in *P. c. chabaudi* AS and CB infected mice, we generated transgenic parasites, PccASluc_230p_ and PccCBluc_230p_, expressing luciferase constitutively throughout the *Plasmodium* life cycle under the control of *eef1a* promoter (Supplementary Fig. 8). At day 4, 6 and 9 dpi, the total parasite load was determined by measuring luciferase activity from 2 μL tail blood when the parasites were at late trophozoite stage^8^, and the level of sequestration in different organs was investigated during schizogony. Consistent with the peripheral load of iRBC (Fig. 1b), there were no significant differences in luciferase activity between PccASluc_230p_ and PccCBluc_230p_ infected mice at 6 dpi either in peripheral blood or by whole body imaging (Fig. 7b). After intensive systemic perfusion, the luciferase activities in isolated organs were measured and relative ratio of sequestration was calculated as the level of luciferase activity per organ (total flux per second) relative to the total parasite load measured in peripheral blood before schizogony (relative light unit, RLU) (Fig. 7c). Consistent with previous findings^8^, both AS and CB iRBC, sequester/accumulate mainly in the spleen, liver and lungs, with no significant signals observed in the kidney or brain. The relative levels of sequestration/accumulation in the spleen and liver were similar between AS and CB infections. By contrast, a significantly higher level of sequestration in the lungs occurred in the CB infection at 6 dpi, and the trend was still maintained at 9 dpi (Fig. 7c). The higher level of sequestration/accumulation of schizonts in the lungs is consistent with the observation of greater amounts of Hz in the lungs of CB infected mice compared to AS infected mice (Fig. 7a).

## Discussion

Host genetics and immune status play important parts in the outcome of an infection with *Plasmodium*^9^,^10^. However, there is an increasing amount of evidence showing that genetic diversity of the parasite also contributes to the varying severity of malarial disease. In this study we used a top-down systems analysis of peripheral blood to investigate whether transcriptomic signatures could be identified that would indicate or predict severity of acute blood-stage malaria caused by 2 strains of *P. c. chabaudi* of differing virulence. Using high-resolution profiling of the whole blood transcriptomics over multiple time points during the acute phase of infection, and data-driven modular analysis, we investigated the involvement of biological processes rather than specific genes, and uncovered several transcriptomic signatures related to severe pathologies in the virulent CB infection. These include distinct signatures for platelet aggregation, anemia and lung inflammation, which can be seen at different time points and distinguished the two infections. This analysis also revealed several signatures common between avirulent AS and virulent CB infections, but they occurred at different time points or were of different magnitude. This highlights the value of studying pathological factors in the host induced by parasites over the course of the infection and not at a single time point.

The platelet aggregation signature was highly up-regulated in all CB infected mice that had reached humane end points. This was the earliest pathology signature identified in this study and similar to the anemia signature was detected before the onset of severe disease. This set of genes was up-regulated as early as 2 days post infection in all CB infected mice regardless of eventual survival. It has been shown that in severe *P. falciparum* infections, platelets mediate iRBC clumping and adhesion^11^,^12^. These observations of association between infection severity and platelets aggregation suggest that similar mechanisms underlie pathology in both the *P. c. chabaudi* model of malaria and in human infections, and the experimental model may be useful to explore the underlying mechanisms. It would be of great interest to analyse the platelet aggregation signature in human malarial infections and investigate whether this transcriptomic signature could be used as an early marker to predict development of severe pathology.

The anemia signature identified was present in the whole blood transcriptome ahead of the clinical onset of RBC loss in both avirulent AS and virulent CB infections, but it was stronger and lasted longer in the CB infection. This transcriptomic signature predicted the more severe and longer-lasting anemia we have observed in CB infections^4^. Both the anemia signature and the heme biosynthesis II pathway analysis indicate a deregulated or stressed host erythropoietic response in the more severe CB infection.

In addition to the platelet aggregation and anemia signatures, we identified a lung inflammatory signature in CB infected mice. Although sequestration of *P. c. chabaudi* AS parasites in lungs has been documented^8^, lung damage has not been previously reported for this experimental model. We confirmed that this SARS-related lung inflammation signature in the blood was indeed associated with a more severe pulmonary neutrophilic infiltration and more cell death in the lungs in CB infections. Furthermore, it was linked to a higher level of sequestration of CB iRBC in this organ. Both *P. falciparum* and *P. vivax* can sequester within the pulmonary microvasculature and cause lethal malaria-associated acute respiratory distress syndrome (MA-ARDS)^13^. Members of the PfEMP1 family (*P. falciparum* Erythocyte Membrane Protein-1) of variant surface-expressed parasite proteins have been shown as parasite ligands mediating parasite cytoadherence^14^. Although PfEMP1 is lacking in other *Plasmodium* parasites, another multigene family PIR (*Plasmodium* interspersed repeat) is present in most, if not all, species of *Plasmodium*; and there is evidence that some PIRs in *P. vivax* bind to ICAM-1 endothelial receptor *in vitro*^15^. It is possible that differential PIRs expression between *P. c. chabaudi* AS and CB is responsible for this differential pulmonary sequestration ability. However, it is also possible that AS parasite is removed more effectively from the lung than CB due to the higher inflammation caused by CB infection.

The higher level of CB pulmonary sequestration leaves greater amounts of hemozoin compared with the AS parasite. There is evidence that Hz can directly induce pulmonary proinflammatory responses^16^. In addition it has been shown that parasite-derived microparticles can induce macrophage activation in a TLR4 (Toll-like receptor 4)-MyD88 dependent manner^17^. In our study, CB infection induced higher level of inflammation (IFNg, IL-6 and MRP14) in the lungs of infected mice. MRP14 (S100A9) is one of the top up-regulated genes identified in the lung inflammation signagure. Together with S100A8 (MRP8), MRP8/14 forms a heterodimer complex that has previously been shown to be a potent chemotactic factor for myeloid cells, especially neutrophils^18^. MRP8/14 are TLR4 ligands and are recognized as damage-associated molecular pattern molecules (DAMP) involved in many inflammatory diseases and infections^19^. For example, in tuberculosis and influenza infection, MRP8/14 is shown to exacerbate pro-inflammatory responses, cell-death and pathogenesis^20^,^21^. Of relevance here, MRP14 protein is significantly increased in *P. falciparum* and *P. vivax* infected patients^22^,^23^. Interestingly, in our *P. c. chabaudi* mouse model, MRP14 was detectable in all mice infected with the virulent CB strain; by contrast, it was detectable in only 40% of mice infected with the avirulent AS strain. Moreover, when MRP14 was detected in AS infected mice, it was present at significantly lower level than that in CB infected mice. This coincided with a significantly higher number of MRP14^hi^Ly6G^+^ neutrophils in the lungs of CB infected mice. It is possible that MRP14^+^ cells respond to the microparticles upon rupture of sequestered CB schizonts, leading to proinflammatory response and recruiting more MRP14^+^ neutrophils. Our analysis offers evidence that different parasite strains, exhibiting different sequestration tendencies, can lead to different levels of lung inflammation and damage.

Deciphering the complex host immune responses during acute malaria is extremely challenging. Here we demonstrate that whole blood transcriptomic signatures can help to reveal severe malaria-associated pathologies, often preceding clinical observations. Our data demonstrate the potential in searching further transcriptomic signatures in human malaria for severity diagnosis and prognosis. Furthermore, these blood signatures can also provide crucial information about the pathogenic processes taking place in organs or tissues during infection, as demonstrated here with the neutrophil-related lung inflammation signature. This unbiased modular analysis of blood transcriptomic data also offers a promising method to search for protective mechanisms in mouse and human malarial infections. This is particularly important for *P. vivax* infections of humans, because of its greater genetic diversity^24^, and the recent surge in reports of severe and fatal *P. vivax* malaria^25^,^26^.

## Methods

### Mice

Female C57BL/6 aged 6–8 weeks from the SPF unit at the Francis Crick Institute Mill Hill Laboratory were housed under reverse light conditions (light 19.00–07.00, dark 07.00–19.00 GMT) at 20–22 °C, and had continuous access to mouse breeder diet and water. Core body temperature was measured with an infrared surface thermometer (Fluke); body weight was calculated relative to a baseline measurement taken before infection; and erythrocyte density was determined on a VetScan HM5 haematology system (Abaxis). This study was carried out in accordance with the UK Animals (Scientific Procedures) Act 1986 (Home Office licence 80/2538 and 70/8326), and was approved by The Francis Crick Institute Ethical Committee.

### Parasites

Cloned lines of *Plasmodium chabaudi chabaudi* AS and CB were originally obtained from David Walliker, University of Edinburgh, UK and subsequently passaged through mice by injection of infected red blood cells (iRBC) at the MRC National Institute for Medical Research, UK and cryopreserved as described^27^. For experimental work, infections were initiated by intraperitoneal (i.p.) injection of 10^5^ iRBC derived from cryopreserved stocks. The course of infection was monitored on Giemsa-stained thin blood films by enumerating the percentage of RBC infected with asexual parasites (parasitemia). The limit of detection for patent parasitemia was 0.01% infected erythrocytes.

Mice were culled upon reaching humane end points by showing the following signs: emaciation (more than 25% weight loss), persistent labored breathing, severe hypothermia (body temperature below 28 °C), inability to remain upright when conscious or lack of natural functions, or continuous convulsions lasting more than 5 min.

*P. c. chabaudi* AS and CB expressing luciferase under the control of the constitutive promoter *eef1a* were generated by transfection with the construct pPc-LUC230p, targeting the neutral *P230p* locus (PCHAS_0308200 or PCHCB_0308200). Transfection and cloning of transgenic *P. chabaudi* parasites were performed as described previously^28^, and integration was verified by Southern blot analysis of chromosomes separated by pulsed field gel (PFG) as described^29^. The construct pPc-LUC230p was modified from pPc-LUCCAM^8^ by replacing *the P. chabaudi* SSU targeting region with *230p* targeting region, Chab03 277001-278950, generated by gene synthesis (Genewiz LLC, NJ, USA).

### RNA isolation and preparation for microarray analysis

Female C57BL/6 mice aged between 6–8 weeks were intraperitoneally infected with 10^5^ iRBC of *P. c. chabaudi* AS or CB. At 2, 4, 6, 8, 10 and 12 days post infection, 0.5 mL of blood was collected via cardiac puncture into 1 mL Tempus RNA stabilising solution (Applied Biosystems). Naïve samples were also collected at day 0 (the day of infection) and day 12 (the end of the experiment) and used as controls. Samples were snap frozen on dry ice and stored at −80 °C until RNA isolation.

Total blood RNA was extracted using PerfectPure RNA Blood Kit (5 PRIME) and GLOBINclear kit (Ambion) was used to remove globin mRNA according to the manufacturer’s instructions. cRNA samples were prepared from 300 μg globin reduced blood RNA using Illumina TotalPrep RNA Amplification Kit (Ambion) and hybridized to Illumina Mouse WG-6 v2.0 Beadarrays according to the manufacturer’s protocols. At each step, the quantity and quality of the RNA samples was verified using NanoDrop 1000 Spectrophotometer (Thermo Fisher Scientific) and Caliper LabChip GX (Caliper Life Sciences).

### Microarray data analysis

Illumina BeadStudio/GenomeStudio software was used to subtract background and scale average samples’ signal intensity and GeneSpring GX 13.1 software (Aigent Technologies) was used to perform further normalization and analyses as described previously^30^. First, all signal intensity values less than ten fluorescent units were set to equal ten, log 2 transformed and per chip normalised using 75^th^ percentile shift algorithm. Transcripts were further normalized to the median across all samples or to the median of control samples. Transcripts were first selected if they were present (cut off 0.99) in ≥10% of all samples, and further filtered with a minimum of 2-fold expression up- or down-change compared with the median intensity across all samples. All microarray data are deposited in GEO under accession number GSE93631.

GeneSpring software was used to perform statistical tests, ANOVA unequal variance with post-hoc Tukey’s HSD (honest significant difference) test, followed by Benjamini-Hochberg multiple test correction (FDR < 0.01); fold change was further performed on the combined list of transcripts differentially expressed either in AS or CB infection, with 2-fold cut off compared to naïve controls. The set of transcripts was defined as differentially expressed transcripts and was used for further analyses. Hierarchical clustering of samples at different infected time points compared to naïve were performed using Pearson uncentred correlation with an average-linkage-clustering algorithm that organizes all transcripts according to their trend of expression across all samples. The Hierarchical clustering on the 6226 transcripts differentiall expressed in AS or CB (Supplementray Table 1) across the acute phase of infection was performed using Euclidean distance metric with Ward’s linkage rule.

For the pathological modules (in Fig. 4), prediction was performed on the 6321 transcripts differentially expressed in AS or CB infection including the samples collected at 9 dpi (Supplementray Table 2) using the self-organising map algorithm in GeneSpring. Euclidean distance was used for similarity measurement and the maximum number of iterations was set at 10e5. The initial learning rate was set at 0.03 and initial neighbourhood radius 5, the number of grids was tested from 10 × 10, till less than 15% of clusters have similarities above 90%, at a final grid of 4 × 6 (24 clusters).

Ingenuity Pathways Analysis (IPA) (Qiagen) was used to identify enriched disease and functions and networks. The significance of the association between the dataset and each analysis was measured using Fisher’s exact test, Z score cut-off 2 and/or P value cut-off 0.01. This program was also used to map the canonical pathways and overlay it with expression data from the dataset. Module annotation was determined using Disease and Function Analysis in IPA.

### IgM quantification in bronchoalveolar lavage fluid

To obtain bronchoalveolar lavage fluid (BALF), mice were terminally anaethetised, and lungs were cannulated and inflated with 500 μL PBS. The liquid was retrieved and spun at 300 *g* for 10 min at 4 °C. Supernatants were obtained and kept at −80 °C till further analysis.

IgM levels in BALF were quantified using a sandwich ELISA. (Southern Biotech).

### Detection of MRP14 and cytokines in sera and lung lysate

Mouse serum was collected via cardiac puncture, clotted in room temperature for 30 min and collected by centrifuging twice at 10,000 *g* for 10 min at 4 °C. Lung proteins were extracted in RIPA lysis buffer with Protease Inhibitor Cocktails (Sigma) and homogenized with POLYTRON homogenizer (Kinematica) on ice. Protein levels were quantified by Pierce BCA protein assay (Thermo Scientific) as per the manufacturer’s instructions. All plates were read on a Safire II plate reader (Tecan).

Mouse S100A9 ELISA kit (R&D systems) was used to determine the level of S100A9/MRP14 in serum and lung lysate samples following manufacturer’s instructions. Cytokine concentrations were determined using Cytometric Bead Array (Biolegend) following the manufacturer’s manual.

### Histology and immunohistochemical analyses

The lungs were extensively perfused with 10 mL PBS and then inflated by injection of 3 ml of 10% neutral buffered formalin (NBF) through the tracheal cannula. Tissue was then fixed overnight in 10% NBF, and transferred into 70% ethanol until embedded in paraffin and sectioned. Each lung specimen was stained with haematoxylin and eosin (H&E). For each mouse, the number of hemozoin crystals were quantified from 10 randomly selected fields on H&E stained slides under Leica light microscopy (400×).

Immunohistochemical staining was performed to examine the expression of MRP14 on paraffin-embedded lung sections with anti-MRP14 antibody (clone 2b10). TUNEL staining was performed using ApopTag^®^ Fluorescein *In Situ* Apoptosis Detection Kit (Merck Millipore) following the manufacturer’s protocol. Imaging of slides was performed on a VS120 slide scanner (Olympus) with a VC50 camera, a UPLSAPO lens, at a magnification of 20× or 40×. Images were processed and analysed using OlyVia Image Viewer 2.6 (Olympus) and Fiji^31^. And TUNEL positive cell numbers were quantified in an area of 1nm^2^ using OlyVia Image Viewer 2.6.

### Flow cytometry

Leukocytes from the lungs were enumerated using flow cytometry. Lungs were excised from mice, diced and digested with 40 μg/ml Liberase (Roche Diagnostics) in Iscove’s modified Dulbecco’s medium (IMDM, Sigma) and then passed through a 70-μm cell strainer (BD Bioscience) and washed with IMDM with 5% FCS. RBCs were lysed for 5 min in RBC lysis buffer (Sigma). Cell counts were performed on a Brightline hemocytometer (Hausser Scientific) with trypan blue exclusion. Cells were seeded in 96-well U-bottom plates and incubated with Fc block (BD), followed by staining with Zombie UV™ Fixable Viability kit (Biolegend) and fluorochrome-labelled antibodies (Biolegend unless otherwise specified): BUV395-cojugated anti-CD8 (BD), BV785-conjugated anti-I-A/I-E, BV711-conjugated anti-CD64, BV650-conjugated anti-CD11c, BV510-conjugated anti-CD3, BV421-conjugated anti-NK1.1, PerCpCy5.5-conjugated anti-Ly6C, FITC-conjugated anti-CD4, PECy7-conjugated anti-CD11b, PE-Texas Red-conjugated anti-F4/80, PE-conjugated anti-Siglec F, APCCy7-conjugated anti-CD19, AF700-conjugated anti-Ly6G, AF467-conjugated anti-MRP14 (BD). Cells were washed with PBS and assessed using Fortessa X20 (BD). Analysis was performed on FlowJo (Treestar).

### *In vivo* imaging and luciferase assay

Mice were infected intraperitoneally with 10^5^ RBC infected with PccASluc_230p_ or PccCBluc_230p_ parasites; and at each time point 2 μL of heparinized tail blood was collected before sequestration^8^. Bioluminescence was assessed with the Luciferase Assay System (Promega) according to the manufacturer’s protocol and quantified with the TECAN Safire2 plate reader and Magellan software (Tecan). Under these conditions, bioluminescence intensity is proportional to the amount of parasites in this blood volume^8^, which reflects the total parasite load before sequestration. At the time of maximum sequestration (12.00–14.00 h GMT, reverse light)^8^, D-luciferin (150 mg/kg, Caliper Life Sciences) was injected subcutaneously 5 min before whole-body and organ imaging. Mice were terminally anaesthetized and systemically perfused by intracardiac injection of 10 mL PBS^8^. The brain, lungs, liver, spleen, left kidney and gut were removed immediately and luciferase assessed using *in vivo* Imaging System IVIS Lumina (Xenogen), with a 10 cm field of view, a binning factor of 4, and an exposure time of 10 s. Bioluminescence (Total flux per second) was quantified with the software Living Image (Xenogen) by adjusting a region of interest to the shape of each organ. To account for the influence of total parasite load on the number of parasites sequestered in the organs, bioluminescence in the organs was normalized to total parasite load. Luciferase activities measured in the organs were divided by parasite load in 2 μl blood (see above), allowing comparison between mice with different parasite burdens.

### Graphs and statistical analyses

All bar charts, dot plots and statistical analyses presented in the figures were made in GraphPad Prism, each dot represents an individual biological replicate and p-values were derived from Mann Whitney U test or multiple T-test.

## Additional Information

**How to cite this article**: Lin, J.-w. *et al*. Signatures of malaria-associated pathology revealed by high-resolution whole-blood transcriptomics in a rodent model of malaria. *Sci. Rep.* **7**, 41722; doi: 10.1038/srep41722 (2017).

**Publisher's note:** Springer Nature remains neutral with regard to jurisdictional claims in published maps and institutional affiliations.

## Supplementary Material

Supplementary Figures

Supplementary Table 1

Supplementary Table 2

Supplementary Table 3
