# Supplementary material for: Signatures of malaria-associated pathology revealed by high-resolution whole-blood transcriptomics in a rodent model of malaria
Source: Sci Rep. 2017 Feb 3;7:41722. doi: 10.1038/srep41722 (PMC5290525; doi:10.1038/srep41722)
Supplement: Supplementary Figures [file srep41722-s1.pdf]

Signatures of malaria-associated pathology revealed by high-resolution whole-blood transcriptomics in a rodent model of malaria

Jing-wen Lin[1*], Jan Sodenkamp[1ƚ], Deirdre Cunningham[1ƚ], Katrien Deroost[1], Tshibuayi Christine Tshitenge[1≠], Sarah McLaughlin[1], Tracey J. Lamb[1‡], Bradley Spencer-Dene[2], Caroline Hosking[1], Jai Ramesar[3], Chris J. Janse[3], Christine Graham[4], Anne O'Garra[4], Jean Langhorne[1*]

[1]Malaria Immunology laboratory, [2] Experimental Histopathology, [4] Immunoregulation and Infection laboratory, The Francis Crick Institute, NW1 1AT

[3] Leiden Malaria Research Group, Leiden University Medical Center, Department of Parasitology, 2333 ZA Leiden, The Netherlands

*Corresponding authors: Jingwen Lin and Jean Langhorne, The Francis Crick Institute, 1 Midland Road, London NW1 1AT, Jingwen.lin@crick.ac.uk and jean.langhorne@crick.ac.uk, Tel: +44 203 796 1498;

ƚ These authors contributed equally.

‡ Current address: University of Utah, Department of Pathology, EEJMRB, 15 N Medical Dr. E, Salt Lake City, UT 84112-5650

≠ Current address: Onderstepoort biological products, Research and Development, 100 old Soutpan Road, Onderstepoort 0110, South Africa

Supplementary Figure 1

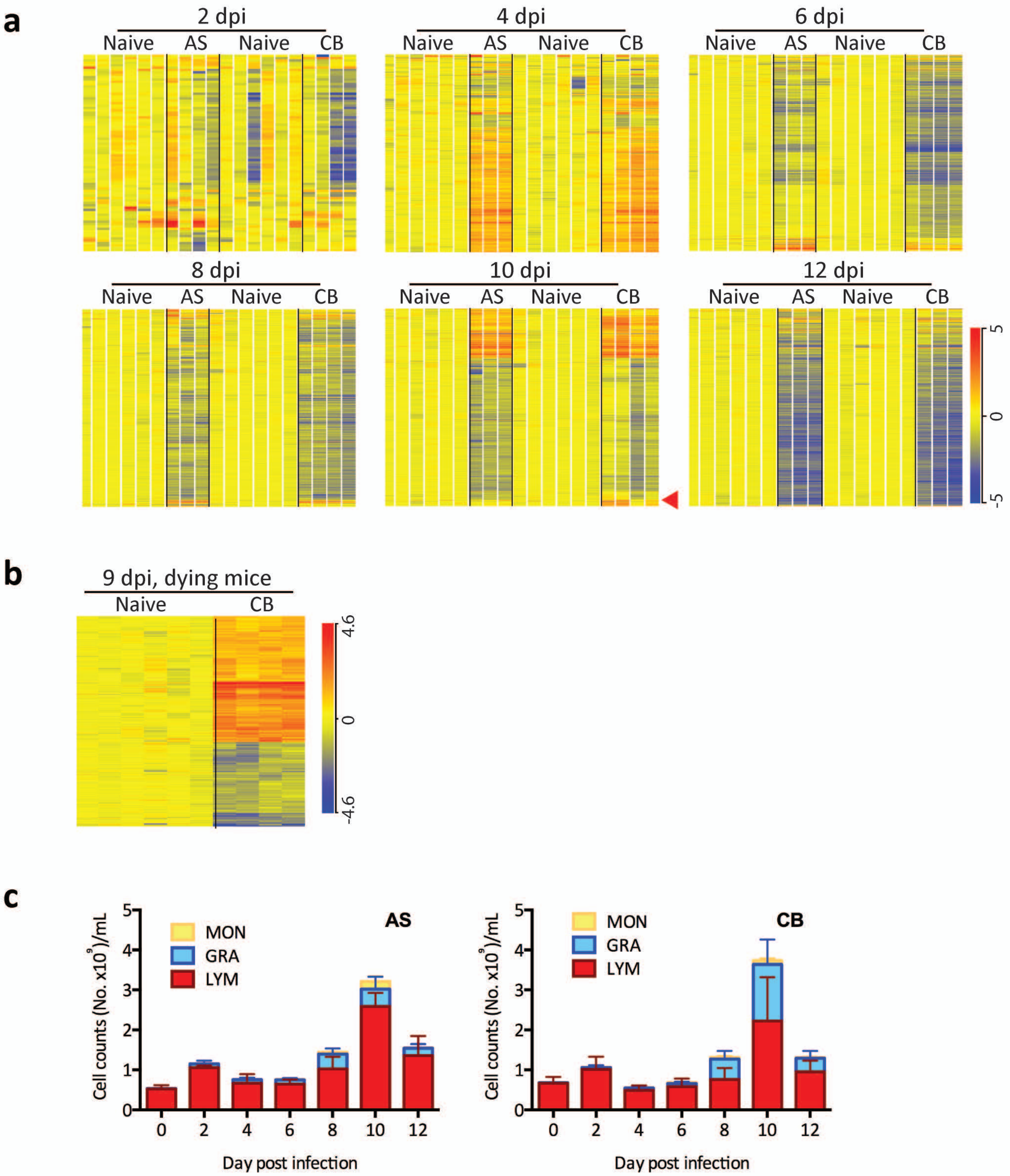

Supplementary Figure 1 | *P. c. chabaudi* avirulent AS and virulent CB strains induce distinct host responses during acute phase of infection.

(a) Hierarchical clustering heatmaps showing differentially expressed transcripts in the blood of mice infected with AS or CB parasites at 2, 4, 6 8,10 and 12 days post-infection (dpi) compared to naïve controls (pooled from naïve samples collected at 0 and 12 dpi). Each column represents an individual mouse (n=3-4), and each row indicates a transcript that is differentially regulated. Red arrowhead shows the transcripts uniquely up-regulated in CB at 10 dpi.

(b) Hierarchical clustering heatmap showing differentially expressed transcripts in 4 CB infected mice that had reached human end points at 9 dpi. Colour indicates log 2 transformed normalized expression intensity in (**a**) and (**b**).

(c) Leukocyte numbers in the blood of AS and CB infected mice compared to naïve mice (day 0) determined using VetScan. MON, monocytes; GRA, granulocytes; LYM, lymphocytes. Mean and SEM are shown (n=4).

Supplementary Figure 2

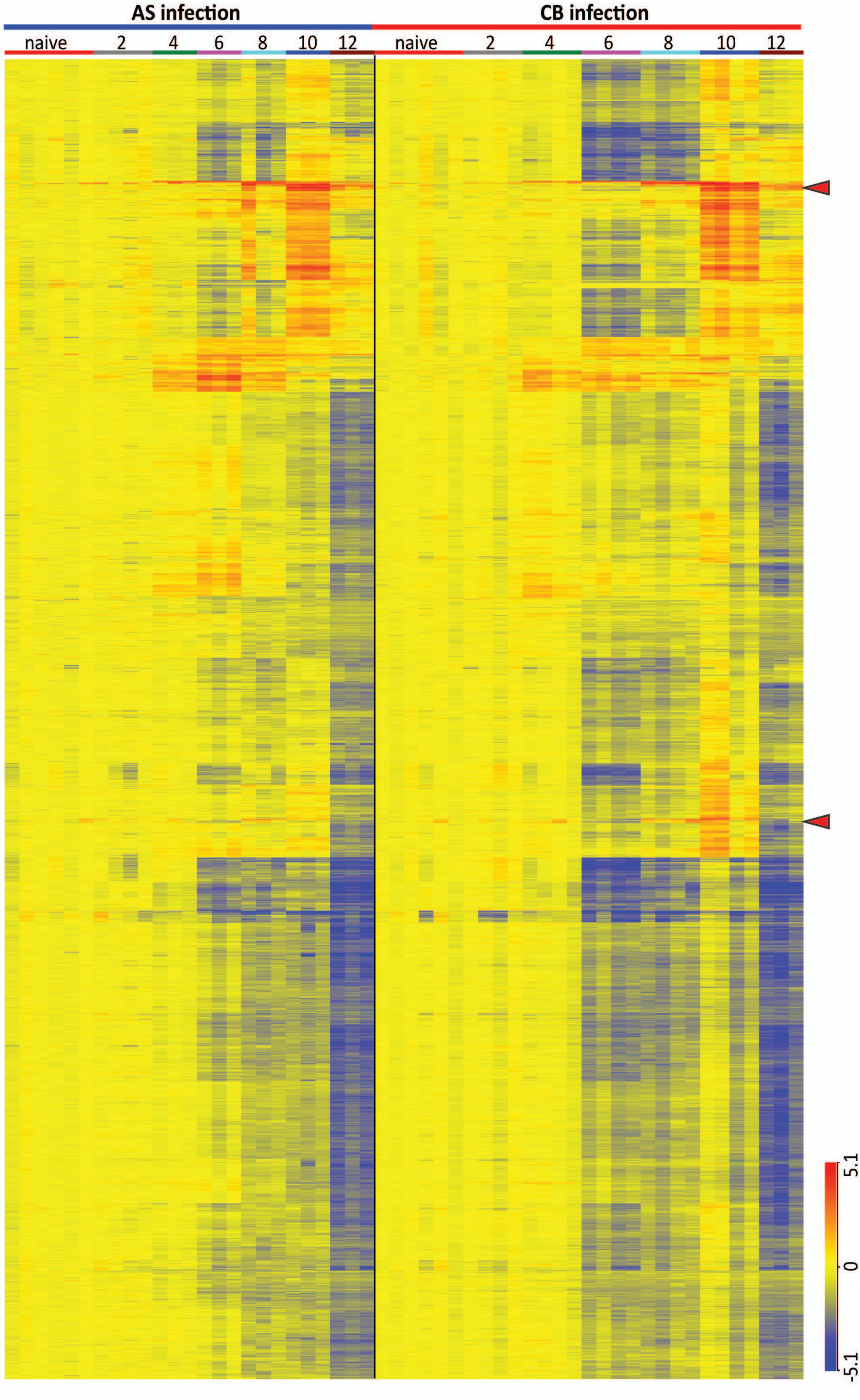

Supplementary Figure 2 | (related to Figure 3a).

Hierarchical clustering heatmap on 6226 differentially expressed transcripts revealed 2 clusters (red arrowheads) comprising 22 genes that were more highly up-regulated in CB than AS infection at 8 and 10 day post infection. Colour indicates log 2 transformed normalized expression intensity.

Supplementary Figure 3

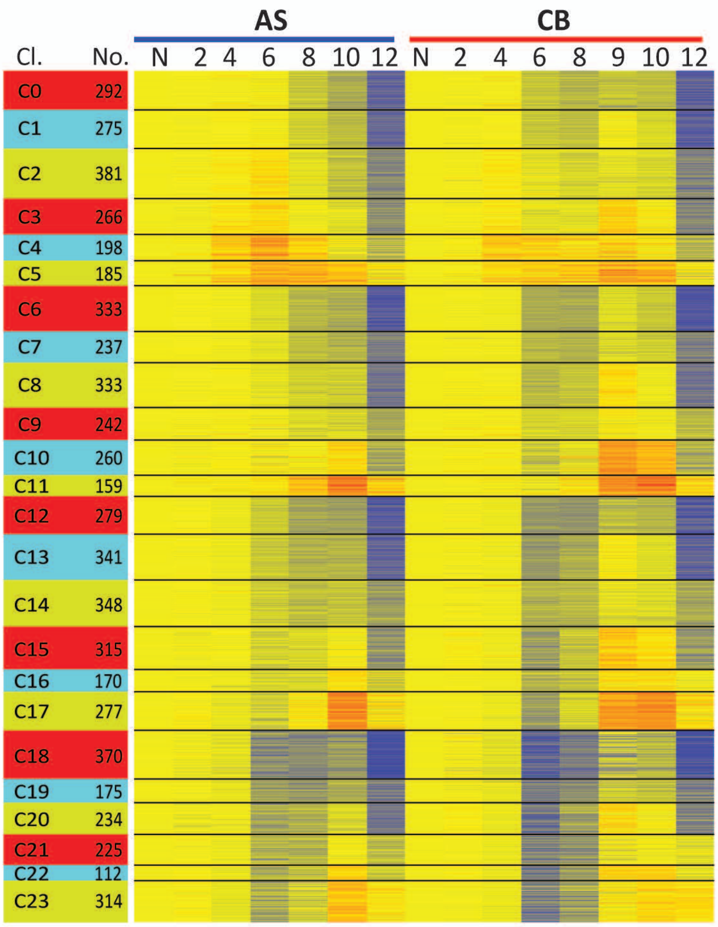

Supplementary Figure 3 | (related to Figure 3d).

Heatmap of twenty-four modules identified by using self-organising map (SOM) method to cluster the 6321 differentially expressed transcripts at different time points in AS and CB infected mice, including 4 CB infected mice that had reached humane end points at 9 dpi. The number of differentially expressed transcripts (No.) in each module was indicated to the right of the cluster number (Cl.). Colour indicates log 2 transformed normalized expression intensity.

Supplementary Figure 4

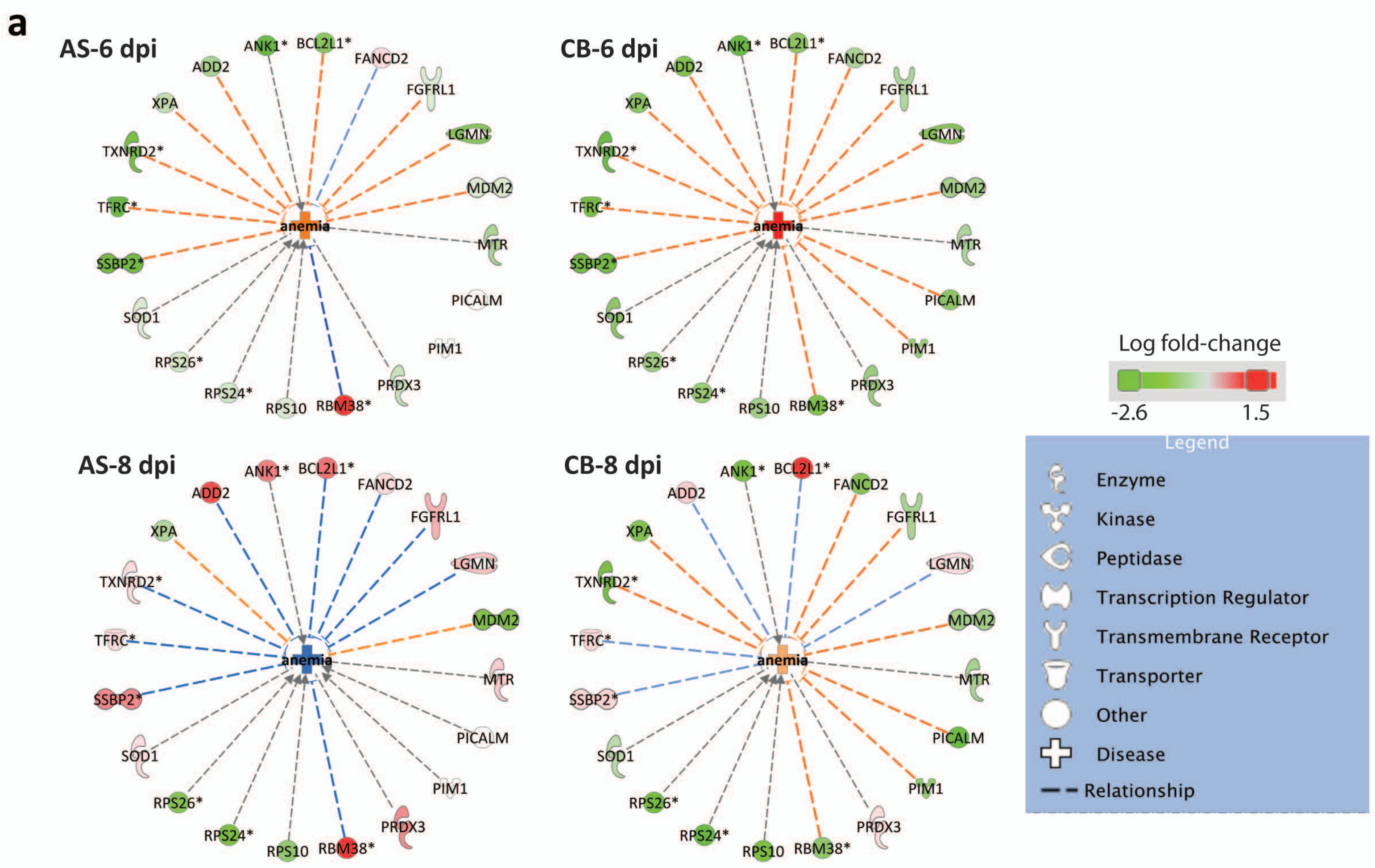

Supplementary Figure 4 | Anemia signature is more elevated in CB infection (related to Figure 5)

Twenty-one genes associated with an anemia signature were more down-regulated in CB infection than AS infection at 6 dpi, predicting more severe anemia in CB infection in IPA. At 8 dpi, most of the genes were up-regulated in AS infection but remained down-regulated in CB infection, predicting alleviation of anemia in AS infection. The relationship between the genes and anemia from IPA disease and function analysis; orange dash lines indicate leading to anemia, blue dashed lines indicate inhibition of anemia, grey lines indicate association with anemia. Colour on the genes indicates log fold change compared to naïve controls. * more than one probe associated with this gene were analysed in the microarray dataset.

Supplementary Figure 5

a Lung inflammation/SARS signature

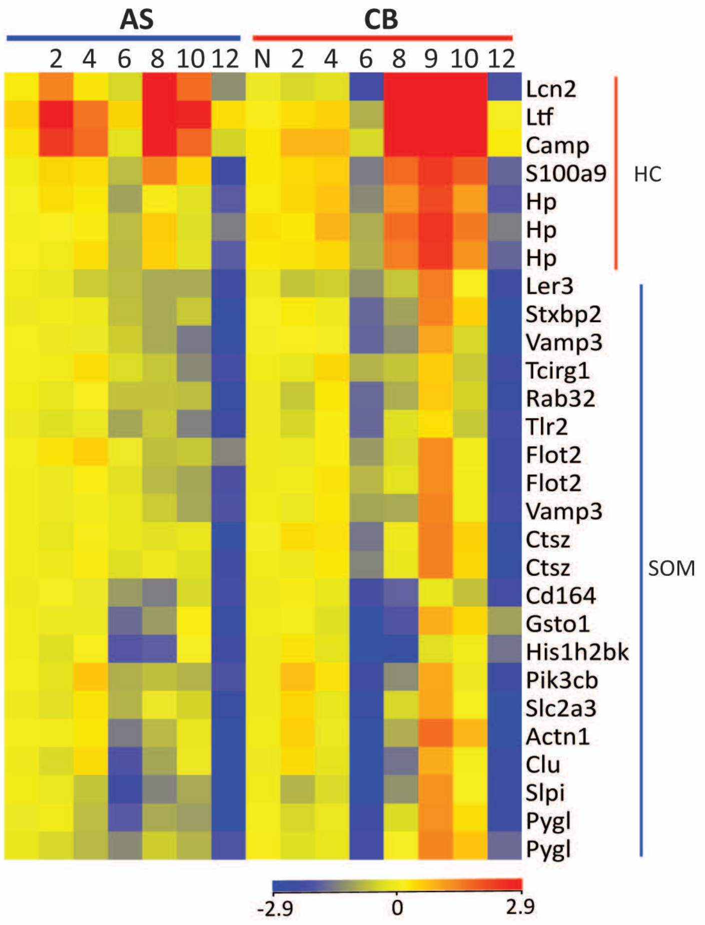

b H&E staininig

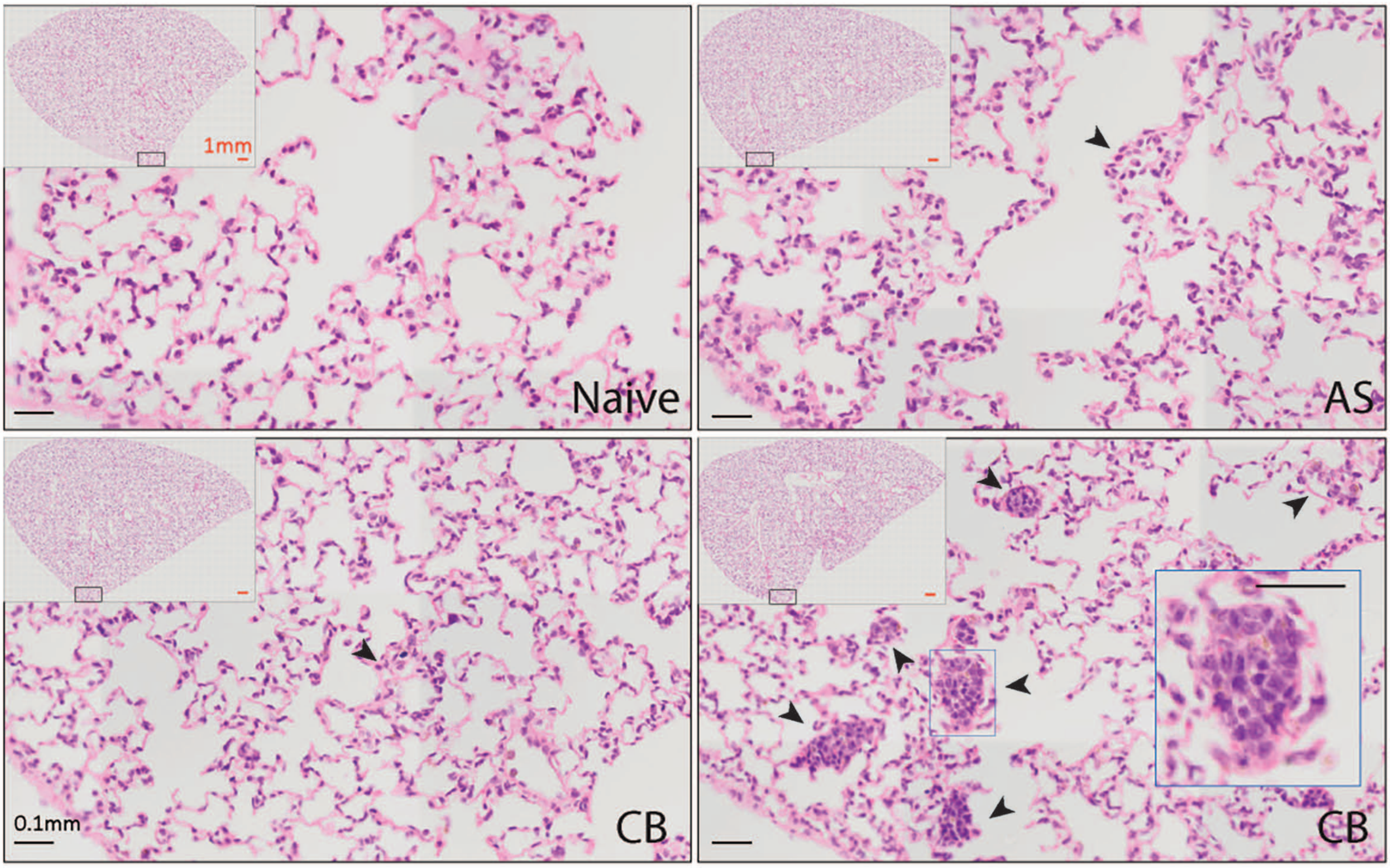

Supplementary Figure 5 | Whole blood transcriptomics analysis revealed lung inflammation signature (related to Figure 6)

(a) A total of 23 genes related to lung inflammation/SARS. Five genes were identified from hierarchical clustering (HC, red shading) and 18 genes were identified from SOM modular analysis (C8 and C20, blue shading). Colour indicates log 2 transformed normalized expression intensity.

(b) Representative images of hematoxylin and eosin stained sections of perfused lungs from naïve, AS and CB infected mice at 9 dpi. Black arrowheads indicate signs of leukocyte infiltration in AS and CB infected lungs.

Supplementary Figure 6

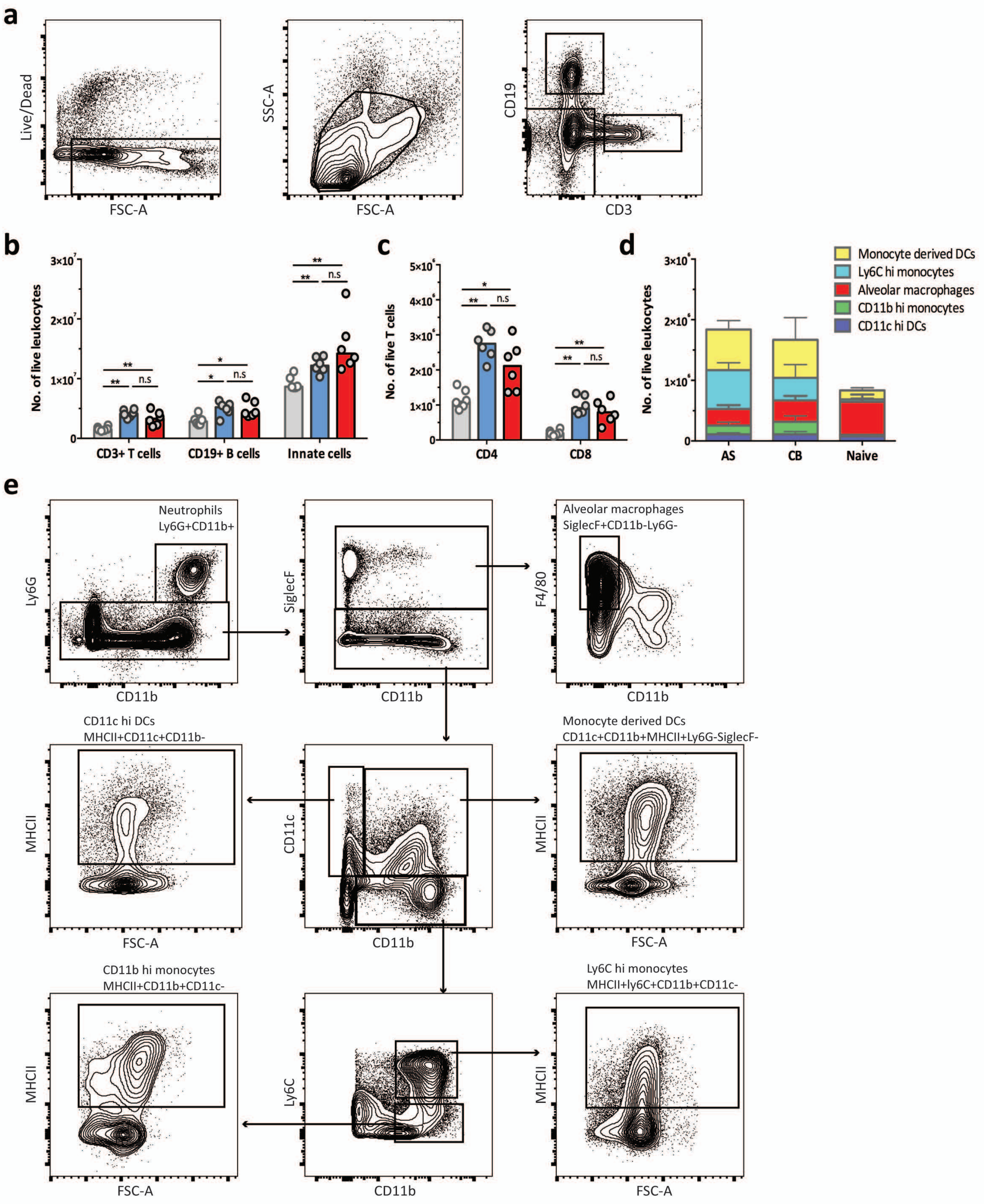

Supplementary Figure 6 | Characterisation of changes in leukocyte populations in the lungs of *P. c. chabaudi* infected mice (related to Figure 6).

(a) Gating strategy of live leukocytes (left and middle panels), and adaptive and innate populations (right panel).

(b-d) Numbers of different leukocyte populations. Median values are shown and each dot represents an individual mouse. Mann-Whitney U test was performed, * $p<0.05$, ** $p<0.005$, n.s, not significant. Data are representative of two independent experiments (n=5-6 per experiment). **(b-c)** grey bars represent naïve mice, blue bars mice infected with AS and red bars mice infected with CB.

(e) Gating strategy and definition of innate cell populations.

Supplementary Figure 7

a

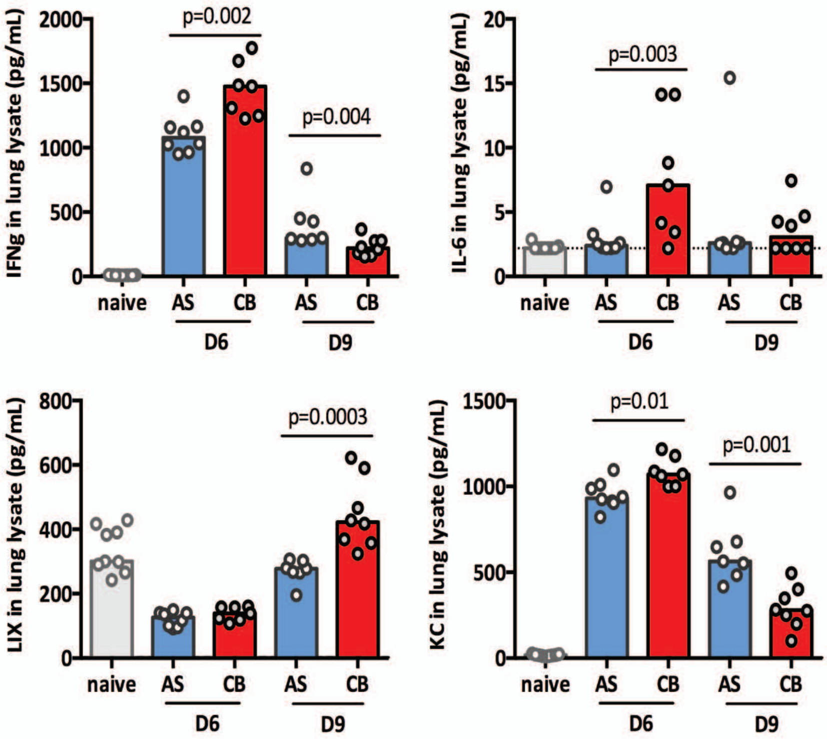

b

Immunohistochemical staining of MRP14

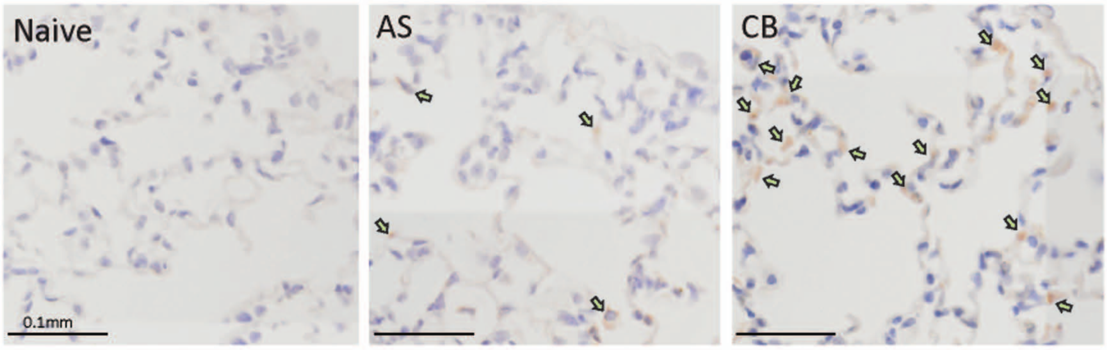

Supplementary Figure 7 | (related to Figure 6).

(a) IFNg, IL6, KC and LIX concentration was quantified by ELISA in lung lysate of naïve, AS and CB infected mice at 6 and 9 dpi. Median values are shown and each dot represents an individual mouse (n>7). Mann-Whitney U test was performed, p values are provided when significance was observed.

(b) Representative images of Immunohistochemical staining of MRP14 on perfused lung sections. Arrows indicate MRP14 positive cells.

Supplementary Figure 8

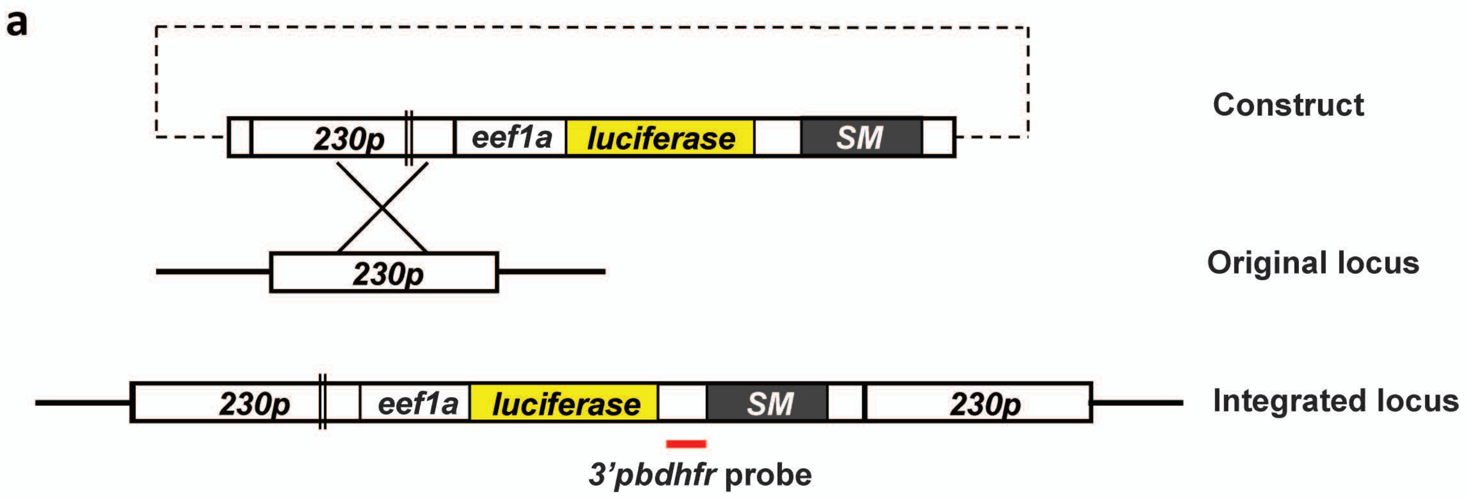

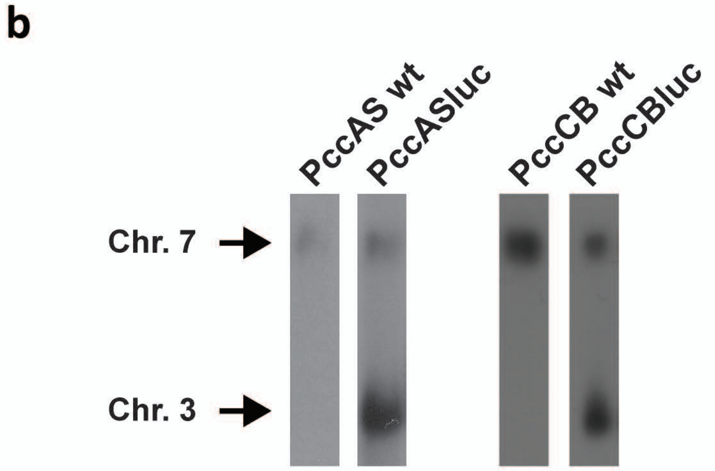

Supplementary Figure 8 | Generation of PccASluc$_{230p}$ and PccCBluc$_{230p}$, expressing luciferase constitutively throughout the life cycle (related to Figure 7).

(a) Schematic representation of the construct containing a luciferase expression cassette, the *230p* genomic locus before and after integration of the construct via single cross-over recombination. SM, selectable marker.

(b) Southern analyses of chromosomes separated by pulsed field gel were hybridised with a probe recognising 3'*pbdhfr* (3'UTR in *P. berghei dhfr* gene) sequence in the luciferase-expressing construct, the location of which was indicated in (**a**). The probe confirmed the integration of luciferase constructs in *230p* on chromosome 3 in both PccASluc$_{230p}$ and PccCBluc$_{230p}$ transgenic lines, and also hybridised at a lower intensity to the endogenous *P. chabaudi dhfr* gene on chromosome 7 in both wild-type (wt) and transgenic lines due to sequence similarities between 3' *pbdhfr* and 3'*pcdhfr*.
